# Medical law and the physician

**DOI:** 10.4103/0970-1591.40602

**Published:** 2008

**Authors:** Nitin S. Kekre

**Affiliations:** Editor, IJU, Department of Urology, Christian Medical College, Vellore - 632 004, Tamilnadu, India. E-mail: editor@indianjurol.com

Is a separate and specific consent for blood transfusion necessary prior to elective surgery? Does consent for surgical procedure automatically means consenting for blood transfusion if required? To illustrate this point let me put forward a hypothetical case. A urologist practicing in a city list a patient for transurethral resection of the prostate (TURP) in a local hospital. He requests for one unit of blood to be cross matched for the procedure. The hospital puts out a request to their regular blood bank. Patient undergoes TURP and has severe intraoperative hemorrhage requiring blood transfusions. Unfortunately the blood was contaminated with human immunodeficiency virus (HIV) and patient contacted HIV infection. Patient then levels allegation against the urologist and the hospital that the blood was transfused without consent and basic safety measures were not adhered to. The urologists and the hospital defense argued that consent for the surgery given by the patient must be interpreted as consent for blood transfusion.

Moreover, blood transfusion is rarely required during TURP and in this situation blood was transfused to save life and consent could not be attempted. As the blood was obtained from a Government approved blood bank, the responsibility of providing safe blood rests entirely with the blood bank. The hospital and urologists cannot be held responsible. Hence there is no negligence on their part and the agrieved party should legally proceed against the blood bank.

The argument appears logical but not legally sustainable. But as often happens just being a good caring doctor does not mean that you have full knowledge of the medical law. A similar type of case (M. Chinnaiyan Vs. Sri. Gokulum Hospital and Anr) has been reported recently in the *Medical Law Cases for Doctors*, Vol 1:1, Special Inaugural Issue, 2008 printed and published by Dr. D.K. Sahu on behalf of Institute of Medicine and Law. This case involved a gynecologist and a local hospital. Patient underwent hysterectomy and required intraoperative transfusions. The gynecologist had requested for blood to be cross matched and such a request was made on the patient's medical chart. Patient contacted HIV. Court found that the gynecologist and the blood bank negligent. Court observed that there were specific directions in the hospital notes for reserving blood. This proved that blood transfusion was anticipated, but no specific consent was obtained. What does this judgment mean to all of us? It simply means that it is safer to obtain separate and specific consent blood transfusion in all cases where blood transfusion is anticipated. As most of the urological procedures do not routinely result in intraoperative or postoperative transfusions, but it may not absolve the urologist from the responsibility of informing the patient about remote possibility of blood transfusion. It would probably be safer to obtain consent for blood transfusion for all procedures. The only exception seems to be emergency (trauma) situation where blood is transfused to save life and consent cannot be attempted. It is of paramount importance that the blood should be obtained from government approved blood banks and it is the responsibility of the hospital to maintain proper protocol to ensure that the concerned blood bank carries out necessary checks and that the blood is accordingly certified. It would be prudent to preserve the copies of the certificate that mandatory testing of the blood has been done by blood bank and should be entered into the medical records of the patient. This case highlights the need for the medical men to be aware of the medical law and a need for continuing legal education. It also highlights that more often we take patients for granted and do not explain them about the surgical procedure and possible complications. How many urologist in our country specifically document and obtain consent for retrograde ejaculation when they perform TURP? Retrograde ejaculation occurs in majority of the patients undergoing TURP. The patient has a right to know that he should be explained about such a complication prior to operation. I am sure that there are many more situations like this and there is a need to evolve a proper system of documentation and consent forms which would avoid potential litigation in future.

Hypospadias is a common congenital anomaly. Historical estimates suggest an incidence of 0.3% in male newborns. Though the recent data from USA suggests modest increase in the incidence, it continues to remain a very challenging reconstructive problem. Though the pendulum seems to have swung in favor of early repairs, the debate between one-stage *vs*. two-stage repairs continue. In this symposium on hypospadias by Dr. Amilal Bhat and colleagues discuss several important issues concerning the care of these unfortunate patients.

Dr. Anita Patel in this issue writes on one of the Doyens of Indian Urology Dr. Karanjawala whose contributions in the field of Pediatric Urology and treatment of Chyluria are very well known. This is the second contribution in the column of legends of urology and I would welcome your feedback. From the next issue, we are planning to start point-counter-point debate on contemporary urological issues.

Last but not the least I welcome Dr. Anil Mandhani, Associate Professor of Urology, SGPGI, Lucknow on the editorial board of *Indian Journal of Urology*.

With best wishes.

